# Biomacromolecules as tools and objects in nanometrology—current challenges and perspectives

**DOI:** 10.1007/s00216-017-0554-9

**Published:** 2017-08-14

**Authors:** Payam Hashemi, Luise Luckau, Petra Mischnick, Sarah Schmidt, Rainer Stosch, Bettina Wünsch

**Affiliations:** 1 Institute for Food Chemistry, Technische Universität Braunschweig, Schleinitzstr. 20, 38106 Braunschweig, Germany; 2 Metrology in Chemistry, Physikalisch-Technische Bundesanstalt, Bundesallee 100, 38116 Braunschweig, Germany; 3 Institute for Physical and Theoretical Chemistry, Braunschweig Integrated Centre of Systems Biology (BRICS), and Laboratory for Emerging Nanometrology (LENA), Technische Universität Braunschweig, 38106 Braunschweig, Germany

**Keywords:** Biomacromolecules, Nanometrology, Proteins, DNA, Polysaccharides

## Abstract

Nucleic acids, proteins, and polysaccharides are the most important classes of biopolymers. The inherent properties of biomacromolecules are contrary to those of well-defined small molecules consequently raising a number of specific challenges which become particularly apparent if biomacromolecules are treated as objects in quantitative analysis. At the same time, their specific functional ability of molecular recognition and self-organization (e.g., enzymes, antibodies, DNA) enables us to make biomacromolecules serving as molecular tools in biochemistry and molecular biology, or as precisely controllable dimensional platforms for nanometrological applications. Given the complexity of biomacromolecules, quantitative analysis is not limited to the measurement of their concentration but also involves the determination of numerous descriptors related to structure, interaction, activity, and function. Among the biomacromolecules, glycans set examples that quantitative characterization is not necessarily directed to the measurement of amount-of-substance concentration but instead involves the determination of relative proportions (molar ratios) of structural features for comparison with theoretical models. This article addresses current activities to combine optical techniques such as Raman spectroscopy with isotope dilution approaches to realize reference measurement procedures for the quantification of protein biomarkers as an alternative to mass spectrometry-based techniques. Furthermore, it is explored how established ID-MS protocols are being modified to make them applicable for quantifying virus proteins to measure the HIV viral load in blood samples. As an example from the class of carbohydrates, the challenges in accurate determination of substitution patterns are outlined and discussed. Finally, it is presented that biomacromolecules can also serve as tools in quantitative measurements of dimensions with an example of DNA origami to generate defined dimensional standards to be used for calibration in super-resolution fluorescence microscopy.

**Graphical abstract:**
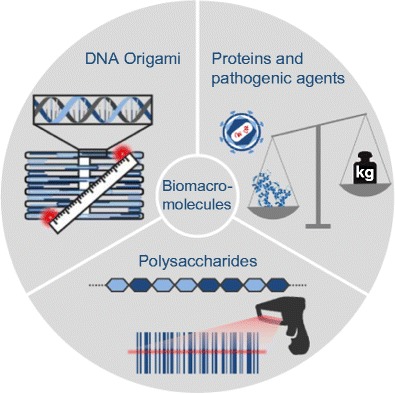
Quantitative analysis of biomacromolecules is accompanied with special challenges different from low molecular weight compounds. In addition, they are not only objects but also tools applicable for quantitative measurements

Quantitative analysis of biomacromolecules is accompanied with special challenges different from low molecular weight compounds. In addition, they are not only objects but also tools applicable for quantitative measurements

## Introduction

Biomacromolecules are naturally occurring macromolecular compounds that make up the essential building blocks of nearly all types of biological systems. Nucleic acids, proteins, and polysaccharides are the most important classes of biopolymers which are formed through condensation of their monomeric units: nucleotides, amino acids, and monosaccharides. The huge number of monomers linked together during polycondensation and the countless combinations that exist for sequencing them lay the foundation for an enormous diversity and structural complexity. Thus, biomacromolecules often exhibit a high degree of functionality and the ability for branching and/or subsequent chemical modification that facilitates the formation of linear and non-linear 2D/3D biopolymer architectures [1].

The inherent properties of biomacromolecules are therefore contrary to those of well-defined small molecules consequently raising several specific challenges which become particularly apparent if biomacromolecules are treated as objects in quantitative analysis. At the same time, this specific functional ability of molecular recognition and self-organization (e.g., enzymes, antibodies, DNA) enables us to make biomacromolecules serving as molecular tools in biochemistry and molecular biology, or as precisely controllable dimensional platforms for nanometrological applications. Beyond that, the ability for molecular interactions and self-organization accompanied by a change of physical properties makes biopolymers a valuable basis for designed biomaterials, e.g., in medicine or for use in controlled drug release. Since the desired properties are also related to the dimensional structure of domains, the components of such functional biomaterials must be thoroughly characterized with respect to topological dimensions.

Given the complexity of biomacromolecules, quantitative analysis is not limited to the measurement of their concentration but also involves the determination of numerous descriptors related to structure, interaction, activity, and function. However, even the determination of the amount-of-substance concentration of a biomacromolecule requires strategies different from those commonly used for the corresponding monomeric constituents.

Several proteins, for instance, can serve as specific early warning indicators of critical body malfunctions related to common widespread diseases like cancer, cardiovascular diseases, or neurodegenerative diseases. The concentration of these protein biomarkers in body fluids like blood serum is, therefore, frequently measured in medical diagnostics. The techniques most widely used in laboratory medicine rely on immunochemical coupling reactions between antibodies and the target proteins realized by means of commercial test kits. SI traceability is realized via primary reference methods [2]. Although established methods like isotope dilution mass spectrometry (ID-MS) have proven to work properly for small molecules, they cannot simply be transferred to whole proteins. The development of reference measurement procedures for large protein biomarkers is therefore an important task in chemical metrology [3].

The dimensions of biological macromolecules typically are within nanometer length scales. Measurement methods used in nanometrology such as SFM, TEM, and SEM are therefore frequently applied for measuring physical, chemical, and dimensional properties of biomacromolecules. On the other hand, nanometer molecular dimensions in connection with their inherent ability of molecular recognition and self-assembly predestine them not only to be objects of analysis but also becoming versatile measuring tools. By the DNA origami technique, custom-shaped regular symmetric structures can be generated [4]. In combination with fluorescent dyes integrated at defined positions, such structures can serve as dimensional reference artifacts, which can potentially be applied for calibrating and certifying nanometrology instrumentation operating beyond the diffraction limit of optical microscopy [5].

In case of the multifunctional and less uniform polysaccharides and their derivatives, we have to consider various types of dispersity: of molecular weight and if applicable of various sugar constituents in the macromolecular chains, of branching and of location of substituents which can only be quantitatively described with probabilities, as patterns and profiles. Among the biomacromolecules, the glycans set examples that quantitative characterization is not necessarily directed to the measurement of amount-of-substance concentration but instead involves the determination of relative proportions (molar ratios) of structural features for comparison with theoretical models, or the average dimension of domains [6, 7].

This paper points out how Raman spectroscopy is currently being combined with isotope dilution approaches for realizing optical techniques as a possible alternative to ID-MS. Furthermore, it addresses current activities aiming at modifying ID-MS protocols to make them applicable for quantifying virus proteins to measure the viral load of HIV in blood samples. As an example from the class of carbohydrates, the challenges in the accurate determination of substitution patterns are outlined and discussed. Finally, it is shown that and how biomacromolecules can also serve as tools in quantitative measurements of dimensions is presented on the example of DNA origami to generate defined dimensional test samples that can be used for calibration in super-resolution fluorescence microscopy.

## Proteins: traceable quantification for detection and monitoring of diseases

Over the last two decades, complex biomolecules such as proteins increasingly gained in importance due to their ability to act as biomarker for supporting early diagnosis and monitoring of widespread diseases. The most commonly used standard techniques for their quantification in human body fluids are immunochemical-based test kits. Nevertheless, the high diversity and complexity of protein markers can lead to varying results depending on the assay design. To assure both SI traceability and comparability of the results of routine laboratory tests, reference measurement procedures are urgently needed.

While most established reference methods in clinical chemistry are based on mass spectrometry [2], recent developments have also demonstrated the capability of optical techniques such as Raman spectroscopy for this purpose [8]. Both techniques unite molecular specificity with the advantages of reliability, accuracy, and repeatability during quantification when applied in conjunction with the concept of isotope dilution (ID). An isotopologue of the analyte, preferably enriched in either ^13^C or ^15^N (or both) serves as an internal standard (spike) which is added to the sample in a known amount. Thus, the molecular mass is the distinctive feature between analyte and spike resulting in signal ratios if mass-sensitive techniques are used for detection. Neither MS nor Raman methods are limited to small molecules as has recently been demonstrated with the example of measuring the total hemoglobin (Hb) concentration in blood, one of the most frequently measured diagnostic parameter in laboratory medicine [9]. This laboratory comparison clearly showed the excellent equivalence of the results obtained with different ID-MS methods and ID-Raman.

Raman spectroscopy provides direct access to structural information of large and diverse molecules such as proteins. Based on molecular vibrations, a mass discrimination between analyte and spike is obtained from a shift of vibrational frequencies while the set of specific vibrations additionally provides a means for unambiguous identification of a compound including possible structural modifications of otherwise similar compounds.

To achieve highest sensitivity, Raman spectroscopy is combined with nanotechnologies resulting in plasmon resonant signal enhancement, known as surface-enhanced Raman scattering (SERS). Coupling of the analyte to suitable metallic nanostructures or nanoparticles does not only improve the detection limits down to the sub μg/g level, but also opens the opportunity for integrating these methods into lab-on-a-chip devices [10]. However, specific detection and SI-traceable quantification of large clinical biomarkers occurring at extremely low concentrations is still challenging. The critical step exceeding the current state-of-the-art is the large increase in molecular complexity as well as the fact that different conformations (isoforms) of the analyte may exist simultaneously and require the use of sophisticated separation techniques. The much larger complexity of biomacromolecules might also mask the isotopically labeled structural sites in the molecule leading to a suppression of those vibrations that are indicative for the discrimination of the two isotopologues. For this reason, alternative approaches to the aforementioned intrinsic labeling and SERS detection schemes have to be considered such as those based on immunochemical antigen-antibody binding [11]. Since restrictions as to the size and complexity of the analytes no longer exist, several modifications of these SERS-based immunoassays for the quantitative measurement of complex molecules such as proteins have been developed [12]. Among the various approaches, extrinsic configurations in which the specific binding of the target biomolecule is detected indirectly from co-functionalization of the nanoparticles with suitable Raman reporter molecules such as DTNB have proven to be particularly suitable for the detection of proteins at extremely low concentrations [13].

**Fig. 1:**
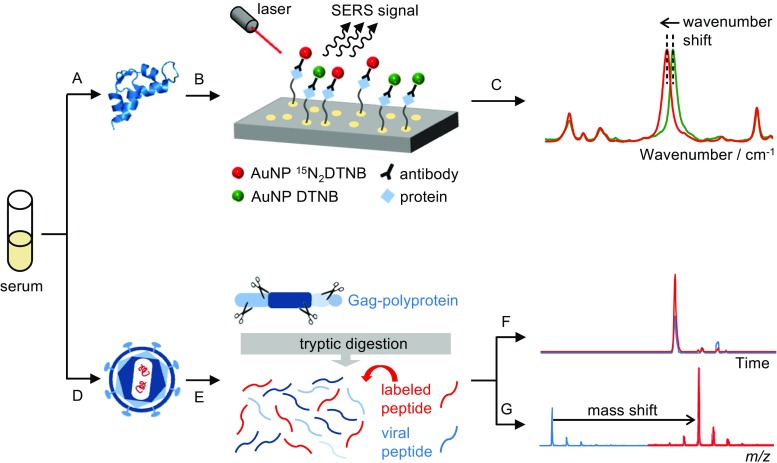
Quantification of serum proteins by isotope dilution surface-enhanced Raman spectroscopy (**A**–**C**) employing isotopically encoded nanoparticles and quantification of virus proteins by isotope dilution mass spectrometry (**D**–**G**)

Further developments towards improved quantitative evaluation of such SERS immunoassays at a metrological level are currently being realized by integration of the ID approach which can be achieved by combination of nanoparticle entities functionalized with the coupling biomolecules and either the native reporter molecule or one of its isotopologues such as ^15^N_2_-DTNB (Fig. 1A–C). Building up the bioassay in this manner provides a suitable basis for realizing ID-SERS independent from the complexity of the protein structure while quantitative evaluation results from evaluating the intensity ratio of the most prominent bands of the two DTNB isotopologues.

Once developed and validated, reference measurement procedures for the quantification of protein biomarkers can also be applied for other diagnostic purposes, e.g., in virus diagnostics. In the case of HIV (human immunodeficiency virus)-infected patients, monitoring of the viral load during therapy monitoring is implemented by the quantification of viral RNA by quantitative real-time PCR assays (qRT-PCR). In these assays, the fluorescent intensities measured at the threshold of the amplification curves are compared with those of a series of external standards with known copy numbers. The latest technological developments in quantitative molecular approaches are digital PCR (dPCR) methods, which enable absolute quantification of infectious agents without external standards. The more accurate quantification of DNA molecules by dPCR is based on the partitioning of DNA at limiting dilution resulting in zero or one molecules per reaction. The capabilities of dPCR for improved reproducible and traceable measurements have been demonstrated in several applications [14, 15]. Nevertheless, for both quantitative molecular techniques (qPCR, dPCR), the RNA extraction step is needed, which contains the major sources of errors because of unpredictable RNA yield and co-extraction of enzyme-inhibiting substances, which can influence the efficiency of reverse transcription and PCR [16]. However, variability is also caused by the usage of different nucleic acid extraction kits and qPCR assays of various designs [17]. Therefore, challenges remain regarding accuracy, comparability, and standardization of measurements because of the lack of primary methodologies and primary reference materials [18].

Consequently, the traceable quantification of viral proteins might be an appropriate alternative approach to overcome present shortcomings in viral molecular PCR tools, and isotope dilution mass spectrometry (ID-MS) would be a further powerful tool in this regard (Fig. 1D). With ID-MS, selected proteins as the HIV gag-polyprotein are quantified via the “bottom-up” proteomics approach by enzymatic cleavages of proteins to specific peptide fragments, which represent fingerprints of proteins and organisms (Fig. 1E). At the beginning of sample preparation, isotopically labeled analogs of peptides or even proteins as internal standard are spiked to the sample (Fig. 1E). The generated peptide mixture is then analyzed by LC-MS. Because of the same chemical characteristics of endogenous and labeled peptides, both elute at the same retention time in chromatography (Fig. 1F) but can be distinguished by their mass differences (Fig. 1G). Especially highly conserved peptide sequences of the HIV gag genome region coding for major structural HIV proteins are suitable for virus quantification. The application of some peptide sequences would allow the quantification of the whole viral load of HIV-1, HIV-2, even though simian immunodeficiency viruses (SIV). The innovation of this method is to define novel prospective measurands such as the determination of the viral maturation level. Newly produced virus particles from infected cells are in an immature status, characterized by the gag-polyprotein-lattice. During the maturation process, this gag-polyprotein is cleaved by the viral protease into the constitutional proteins: matrix, capsid, spacer 1, nucleocapsid, spacer 2 and p6 peptide. Because of the high versatility of the ID-MS approach, it is possible to select peptides, which are present within the individual proteins as capsid or nucleocapsid of the gag-polyprotein and thus throughout the whole maturation process. Such peptides can be used to deduce the amount of the total HIV particles (HIV viral load). In contrast, peptides located between individual proteins of the gag-polyprotein are cleaved during the maturation process and hence are specific for immature virus particles. Combining both parameters finally allows the calculation of the viral maturation level, which would be a novel measurand only available by protein quantification techniques as ID-MS.

Furthermore, the development of independent approaches to virus quantification provides a more detailed knowledge about the correlations between viral protein and RNA amounts and possible discrepancies between these values and measurement failures. In this manner, exact measurements of viral RNA and protein amounts combined with calculations of HIV particle sizes might be used for the development and characterization of certified HIV reference materials which up to now are not available. In addition, the developed and validated ID-MS method can be used as reference for calibration of conventional qPCR assays.

## Polysaccharides: quantifying substituent patterns

In contrast to proteins and DNA with their identical copies of defined macromolecules, polysaccharides—the most abundant class of biopolymers—usually do not possess a distinct size and sequence. Rather, they show various types of dispersity with respect to molecular weight, structure, and chemistry, including branching, sequence, and average composition of constituents. Furthermore, their polyfunctionality invites to chemical modification to obtain new materials, e.g., for biomedical and construction applications. To elucidate structure-property relationships, the structure has to be described in form of quantitative patterns and profiles with the highest resolution possible. As an example, water-solubility and thermoreversible gelation of methyl cellulose (MC) do not only depend on the average degree of substitution (DS) with methyl groups, but also on their location on the cellulose-constituting monomer, glucose, and on their distribution along and over the macromolecules [19]. Consequently, the analytical challenge is not the determination of absolute concentrations of uniform and defined analytes, but the probabilities of certain structural features, defined by their average degree of polymerization (DP) and DS.

**Fig. 2:**
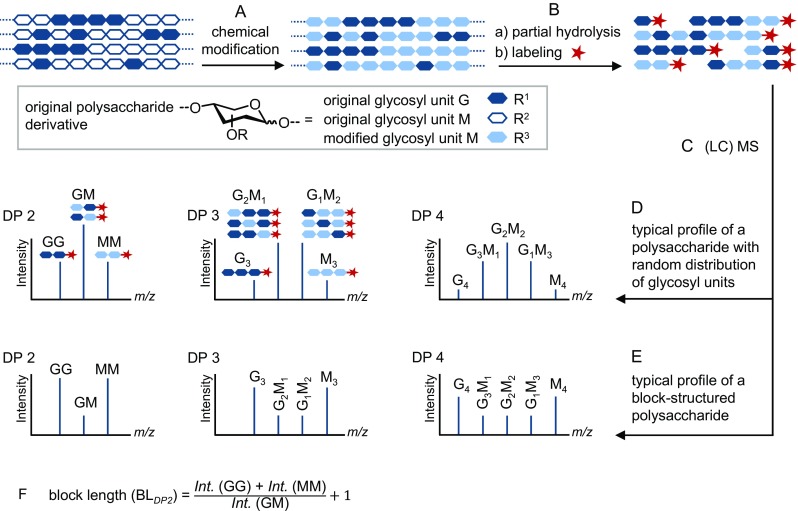
General procedure for modification and quantitative analysis of polysaccharide derivatives by (LC)-MS. Steps **A**–**F** are outlined in the text

Similar analytical challenges are the characterization of methyl esterification pattern in pectins, block length in alginates, sulfation pattern in glycosaminoglycans or carrageenans, or distribution of residual *N*-acetyl groups in chitosan. Beside specific depolymerization, appropriate derivatization and separation techniques, mass spectrometry (MS) is the method of choice for quantitative characterization of these types of complex samples. Due to the sensitivity of soft ionization techniques and mass analyzers to the chemistry and mass of analytes, mixtures of large heterogeneity with respect to DP and substitution pattern profiles cannot be analyzed quantitatively without further modifications (Fig. 2A). Instead of adding isotopomers of the analyte as internal standards, isotopic modification is performed for quantitative MS analyses since isotopically modified derivatives have similar chemical and ionization properties as the unmodified analytes, but are slightly shifted to higher *m/z* in the mass spectra. Due to the wide dispersity of polysaccharide derivatives, it is not possible to have a general modification or analytical method, but each case should be considered individually. Next paragraph shortly outlines basics about sample preparation and quantitative analysis of substituent patterns by MS as it is applicable to glucan ether derivatives.

Correct molar ratios of analytes belonging to the DS profile of a certain DP are the basic requirement for a reliable substituent pattern analysis. To determine the substitution pattern or, where required, block length of multi-block structures [20], depolymerization into shorter oligosaccharides is a necessary step which is usually performed by either partial acid hydrolysis (Fig. 2B), or mild enzymatic degradation in case of polysaccharides bearing sensitive substituents such as ester groups. The selectivity with respect to the kinetics of depolymerization is a source of error due to inductive and field effects of substituents and their influence on solution state and flexibility of chain conformation [21].

In case of partially *O*-methylated glucans, for instance, these effects can be eliminated by full alkylation with deuteromethyl groups, while the original substitution pattern is preserved and detectable by MS. Moreover, discrimination in electrospray ionization (ESI) of analytes caused by different surface activities is overcome by this way of “isotopic labeling” [22]. Such proper chemical modification of analytes is not always possible, and MS analysis can still cause bias. In ESI, matrix effects can cause strong mutual ion suppression [23]. Surface activity, electrophoretic mobility, desolvation energy, cation complexing ability, sample and electrolyte concentration and flow rate of infusion have an impact on ion yield, while instrumental parameters determine the ion transmission with respect to *m/z*. Discrimination due to discrepancies of complexation affinities to sodium are solved by introduction of tags carrying a permanent charge such as Girard’s T or aminobenzoic acid (Fig. 2B). Coupling MS with liquid chromatography (LC) does not only provide separation of analytes before analysis by the mass spectrometer (Fig. 2C), but also enables programming the instrumental parameters to their optimum values during a run [7]. Less competition among analytes might reduce ion suppression and improve signal-to-noise ratio, but at the same time, the compound of interest is detected at different concentrations along the peak profile, which might be critical due to concentration dependence of absolute and relative ion yields. In a recent study, Gangula et al. investigated quantitative analysis of *O*-Me and *O*-Me-*d*_*3*_ glucan ether mixtures by means of ESI-MS [24]. With increasing total concentration of methylated (Me) and deutermethylated-(Me-*d*_*3*_) maltooligosaccharides, the absolute ion intensities increased up to the surface saturation point of droplets generated by electrospray from which absolute intensities remained constant with certain discrepancy. Furthermore, it is possible to improve the quantitative MS analysis by employing an appropriate internal standard addition method, thus neutralizing the effect of out of control measurement conditions [22].

Figure 2 demonstrates a general procedure for modification and quantitative analysis of polysaccharide derivatives by MS. For simplification of the figure, a polysaccharide derivative comprising only two types of glycosyl units (G and M) is depicted. Corresponding peak patterns for a random (D) and a block (E) copolymer are shown, respectively. By replacing the peak intensities acquired from the MS spectra into a simple model (Fig. 2F), it is possible to calculate the average block length (BL) of blocky polysaccharide derivatives at sub-10-nm scales [6].

## DNA: self-organized origami structures as pretest samples for bioimaging

While we have just shown how biomacromolecules can serve as objects, we will now focus on their use as a tool in nanometrology. We therefore discuss DNA origami because they are currently one of the most prominent nanoscopic examples.

DNA is a useful tool in quantitative and qualitative analysis. Due to its simple structure compared to proteins and enzymes, it can be controlled quite well. The most important advantage of DNA is the strong and specific hybridization of two single strands (Watson-Crick base pairing). Depending on the length of the DNA strand and the selection of bases, the strength of the bond between two single-stranded DNA (ssDNA) pieces can be adjusted. The size and properties of double-stranded DNA are well known and can be used for the controlled self-assembly of nanodevices (DNA origami) with very high yields of correctly folded shapes after purification [4]. The synthesis is much cheaper and faster than lithographic fabrication of nanostructures.

**Fig. 3:**
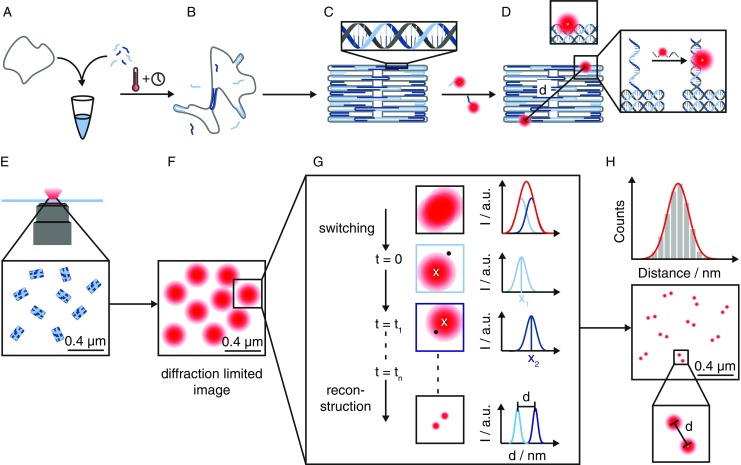
Self-assembly of DNA into a two-dimensional rectangular DNA origami, modified with two dyes at a certain position. Bottom row: Sketch of super-resolution microscopy of DNA origami. Steps A–H are outlined in the text

The DNA origami technique was introduced 2006 by Paul Rothemund [4] and a sketch of the method is illustrated in Fig. 3A–D. In a one-pot reaction, a long and circular DNA single-strand (scaffold) is mixed with an excess of shorter single-strands (staples) (Fig. 3A). The staples each bind to two complementary sequences which are located on different parts of the scaffold and fold it into the desired shape while the sample is heated up (Fig. 3B). When all staples are bound to their complementary part on the scaffold and the sample is cooled to room temperature, the folding is finished (Fig. 3C). In this example, a two-dimensional rectangular DNA origami was created. After purification by gel electrophoresis or filtering, a high number of identical structures (10^6^–10^10^) can be obtained.

Another key aspect of the DNA origami besides the fast and easy assembly of billions of identical structures in one experiment is the easy way of adding dyes, nanoparticles, proteins and other small molecules of interest. Thus, a dye linked to DNA can be inserted into the DNA origami while folding (Fig. 3D top inset), and the DNA strand will bind to a certain position of the scaffold. The other possibility is the extension of an incorporated strand by several bases (12–15) that will point away from the DNA origami. After folding, DNA modified with a dye will bind to the extended DNA strand (Fig. 3D bottom inset). Thus, a so-called DNA origami nanoruler can be designed with a specific distance between two dyes (e.g., 98 nm [25]). An application of DNA origamis as rulers for (optical) microscopy [5, 25, 26] will be discussed in this chapter.

In super-resolution (SR) microscopy distances smaller than the diffraction limit of optical light can be resolved. The most common super-resolution microscopy techniques are structured illumination microscopy (SIM), stimulated emission depletion (STED) and localization-based microscopy (STORM, PALM, SOFI, PAINT). The advantage of fluorescence-based SR microscopy techniques over conventional techniques (e.g., electron microscopy) is the possibility to measure biological structures without their destruction, even in living cells.

Until the concept of DNA origami nanorulers was presented, the resolution limit was testified using, e.g., actin filaments or short dsDNA. The resolution is then determined as the smallest distance resolvable between two actin filaments or two dyes on the dsDNA strand. The drawbacks of actin filaments (unknown number of dyes, random distribution of dyes, and random distances between filaments) can be overcome by the use of dsDNA. Therefore, the DNA origami became an important tool in super-resolution microscopy. With DNA origami nanorulers, a distance between dyes in the range of 6 to 360 nm is achievable and the quantification of the resolution of a microscopy system under ambient conditions is possible.

There are various techniques available for SR microscopy. In the following, we will focus on dSTORM to explain the application of DNA origami nanorulers. In the first step, the DNA origami is immobilized on a glass surface (electrostatically or via protein-protein interactions). By the use of wide-field fluorescence illumination, hundreds of molecules can be observed simultaneously (Fig. 3E). This results in a diffraction-limited image (Fig. 3F). Addition of a specific buffer will make the dyes blink. This way the dyes will fluoresce at different times. Every time a dye is fluorescing its point spread function can be fitted by a Gauss function and the exact position of the dye can be localized (Fig. 3G). After every molecule was detected several times without disturbance of surrounding molecules, a super-resolved image can be constructed (Fig. 3H bottom). By determination of the distances for all DNA origami nanorulers, a Gaussian distribution is obtained which will give the mean distance together with the localization uncertainty (Fig. 3H top). The localization uncertainty Δ*x* is proportional to the inverse square root of the number of emitted photons per localization: $\Delta x\sim \sqrt{N_{photons}}$.

A significant example for the use of DNA origami nanorulers is described by Balzarotti et al. [27]. They tested a novel microscopy setup with DNA origami nanorulers before tracking single 30S ribosomal protein subunits. This shows that there is another analysis method for proteins with super-resolution microscopy. But proteins are not only an object of interest; they can also be a tool in super-resolution microscopy. Jusuk et al. replaced the organic dyes by fluorescent proteins and used them to resolve a DNA origami nanoruler and afterwards microtubules in fixed mammalian Vero cells [28]. Fluorescent proteins have the advantage over organic dyes that they can be expressed in living cells directly and do not have to be inserted into the cell.

All in all, DNA origami nanorulers provide the possibility to measure multiple identical distances at the same time. In addition, the DNA origamis can be easily modified to test for different distances as well as different wavelengths.

## Conclusion and perspectives

The methods and applications discussed in this perspective are developing strategies for the analysis of biomacromolecules at the nanoscale. Quantification of biomacromolecules that serve as diagnostic markers is a challenging task in medical diagnostics. In this context, the availability of reference measurement procedures is of major importance since only those methods are suitable to adequately provide the necessary quality assurance in clinical laboratory medicine. Alternatives to ID-MS-based techniques such as ID-SERS are suitable to complement the portfolio of existing metrological tools. This would in any case be justified, since the huge number, diversity and complexity of potential biomarkers will most probably not be covered by a single universal analytical approach. Further development of existing MS methods towards the determination of viral loads would also help to improve reliability and comparability of routine test procedures for disease monitoring. Both the patients’ well-being and the healthcare system would likewise benefit from the availability of such a metrological basis in clinical chemistry.

In case of complex mixtures of polysaccharide derivatives, mass spectrometry is a promising technique for determination of structural features, e.g., substituent pattern or nanoscale block lengths of blockwise-structured polysaccharide derivatives, provided proper isotopic modification is employed to suppress the chemical and instrumental discriminations. Developing a robust, yet highly accurate method for structural analysis of such complex derivatives provides better understanding of structure-property relationships, and forges the way to preparation of tailor-made materials.

Self-assembled DNA-origami nanorulers are promising nanostructures that serve as a tool for measurements at the nanoscale. They are commonly used in super-resolution microscopy of biological samples to test the resolution under physiological conditions. Due to the huge number of variations that can be applied to control the design of a DNA-origami nanoruler, any possible distance between arbitrary chosen dyes can be realized. A further step in the nanoruler development is to use them in biological samples as a comparison tool for the estimation of nanometer distances.
